# Case of Congenital Fissure of the Soft Palate

**Published:** 1827-02

**Authors:** Herbert Mayo


### FISSURE OF THE SOFT PALATE.

### *Case of Congenital Fissure of the Soft Palate.*

By HERBERT MAYO, Esq. &c.

[WITH AN ENGRAVING.]

GEORGE TUCKER, aged twenty-six, a native of Daventry, applied to me for advice under the following circumstances:—His soft palate had been imperfect from birth; a fissure divided the uvula into two equal portions, and extended to the margin of the palate bone. The soft palate, therefore, consisted of two pendulous flaps, which receded from each other at such an angle that the halves of the uvula were about an inch apart. The appearance of the fissure is represented by fig. 1 of the adjoined Plate.

This patient could neither swallow nor articulate like other persons. Each time that he attempted to swallow a draught of liquid, some of it escaped through the nostrils; and part even of the solid food which he ate made its way through the fissure, so as to rest upon the upper surface of the palate, till he managed to dislodge it. His speech was unpleasant and guttural, not unlike that of a person with an harelip.. He could, however, articulate every letter intelligibly, except the letter T.

On being desired to make the effort of swallowing, with the mouth empty and sufficiently open to allow the soft palate to remain visible, the flaps of which the latter consisted were seen to be drawn forwards and towards each other, so as nearly to meet, in the manner represented by fig. 2 of the adjoined Plate. At the third or fourth repetition of this effort, the flaps were observed not to be drawn so close as at the first. He had been in the habit of remarking that, towards the close of a repast, his deglutition became much less perfect than at its commencement.

Every circumstance promising success, I recommended him to undergo an operation, to which he cheerfully agreed: it was performed on the 8th of last December, with the assistance of Mr. Cooper and Mr. Fieldsend, two gentlemen at present attending the lectures in Great Windmill-street.

The edge of either flap of the soft palate was removed with a thin double-edged scalpel, the part to be removed being fixed by means of Assalini's tenaculum; little more than the membrane covering the edge of each flap was taken away. Four sutures were employed. The ligatures were introduced by means of a small curved needle, fixed in a strong porte-aiguille. The ligatures passed through the middle of the cut surface, and pierced the membrane at the distance of four or five lines from the cut edges. The three upper ligatures were drawn tight, and tied, in succession. The lowest, when drawn tight, produced coughing and a sense of suffocation: it was loosened to relieve these symptoms, and afterwards tied in such a manner as merely to hold the cut surfaces at that part in apposition.

On the evening of the 8th, the part presented a favourable appearance. The patient swallowed the small quantity of liquid food allowed him, without the return of any part by the nostrils.

9th.—The uppermost and the lowest ligatures had produced more ulceration than the others.

10th.—The two uppermost ligatures were removed.

11th.—The remaining ligatures were removed: every part of the line of union appeared to hold.

12th.—The inflammation and soreness had diminished. Parts of the uniting substance, and the holes through which the ligatures were drawn, had a foul appearance, and were touched with balsam of Peru applied on a camel's-hair pencil.

13th.—The general appearance of the part was improved; but a hole, a line in diameter, had dropped through the upper part, and a larger hole appeared immediately above the uvula. The surfaces were touched with a weak solution of lunar caustic.

The hole at the upper part of the line of union closed spontaneously in the course of a few days: that above the uvula continued to enlarge, and in three days the adhesion of the parts of the uvula gave way. Two-thirds, however, of the original fissure had firmly united, presenting the appearance delineated in fig. 3. The patient could now swallow solid food perfectly, and his deglutition of liquids remained much improved; but something had certainly been lost, since the lower part of the adhesion gave way. On his taking a draught of liquid, some drops were again found to escape by the nostrils The patient, notwithstanding, was less disappointed at the partial failure of the operation, than encouraged by its general success to desire its repetition upon the part which had become disunited.

Accordingly, on the 31st of December, in the presence of my friends, Dr. Waring, Mr. Hawkins, and Mr. Rowlands, I removed the edges of the remaining fissure, and sewed it together with two ligatures.

January 1st.—The ligatures had caused considerable ulceration on the left side.

2d.—The upper ligature was removed in the afternoon; and in the evening I took away the lower, which had nearly cut its way through upon the left side. The patient was directed to swallow nothing till the following morning.

3d.—The appearance of the parts was favourable. The adhesion seemed least perfect near the upper angle of the wound.

4th.—A small hole appeared above the uvula, which enlarged in the course of two days to two lines in length and breadth. The surface now became clean, and granulated, but the hole remained stationary.

On the 9th, I added two glasses of port wine to his diet of broth and jelly, and arrow-root; and directed him to hold the wine some time in his mouth, that it might operate as an astringent application upon the part. The hole now began to close rapidly, and by the 14th, when he expressed a wish to return to his family, had contracted to less than a line in diameter; so that it appeared to me needless to detain him the few days longer in town, which would have given me the satisfaction of seeing the part entirely restored.

In fig. 4, the appearance of the soft palate eleven days after the operation is delineated.

He can now swallow perfectly, and is able to articulate his own name distinctly, which he had previously pronounced "Ucker:" the guttural tone, however, in which he speaks remains unchanged, and probably will continue so.

On reviewing the details of this case, I should be inclined to deduce from it the following advice with regard to the management of another ; and, although the rules may appear trivial and obvious, and may be inapplicable to many instances, they are at any rate not likely to mislead, when stated in connexion with the facts which suggest them.

1. A patient should have lived as heartily as usual, or nearly so, up to the day of the operation, in order to bear the prolonged abstinence which is requisite afterwards.

2. On the day of the operation, and on the following, the patient should take a moderate quantity of liquid nourishment.

3. All the ligatures require to be removed by the third day : during the ensuing two, three, or four days, the patient should submit to the most rigorous abstinence, taking no more than a few table-spoonfuls of broth or a melted jelly twice in twenty-four hours.

4. The ligature should consist of a single thread, rendered double by passing through the needle. The ligature should pierce the membrane of the fauces at five lines from the cut edge, and be carried through the middle of the substance of the soft palate.

5. The ligatures should be drawn tight :—if they are merely drawn till the cut surfaces are brought into contact, the ulceration which immediately follows is liable so to loosen them that the first adhesion may be partially dissolved or materially weakened.

6. In the event of a hole appearing in the line of union, no local application should be used till the surface has become clean and granulating.

7. The substance of the soft palate is of great toughness, and requires that the needle employed to perforate it be very firmly fixed in the port-aiguille. But, as soon as the needle is introduced, considerable irritation occurs, rendering it necessary to complete the operation of drawing the ligature through as quickly as possible. For this purpose, the point of the needle may be readily seized with forceps :—but it is not equally easy to disembarrass the port-aiguille of the ring which secures the needle in its socket. A simple contrivance might remedy this defect in the common port-aiguille. A second ring (as represented by fig. 5,) might be added to the instrument, connected to the first by a slide, and having a projection, upon which the thumb might operate with sufficient purchase.

19, *George-street, Hanover-square ; Jan.* 15, 1827.

